# Supplementary material for: Pyruvate Dehydrogenase Kinase 1 inhibition mediated oxidative phosphorylation enhancement in cartilage promotes osteoarthritis progression
Source: BMC Musculoskelet Disord. 2023 Jul 20;24:597. doi: 10.1186/s12891-023-06585-6 (PMC10357736; doi:10.1186/s12891-023-06585-6)
Supplement: Supplementary file 1 — Additional file 1. [file 12891_2023_6585_MOESM1_ESM.zip › MF.Gsea.1653627316242/GOMF_PEPTIDASE_ACTIVATOR_ACTIVITY_INVOLVED_IN_APOPTOTIC_PROCESS.html]

Details for gene set GOMF\_PEPTIDASE\_ACTIVATOR\_ACTIVITY\_INVOLVED\_IN\_APOPTOTIC\_PROCESS[GSEA]

|  || Dataset | 55457\_collapsed\_to\_symbols.cls.cls #OA\_versus\_normal.cls.cls #OA\_versus\_normal\_repos |
| Phenotype | cls.cls#OA\_versus\_normal\_repos |
| Upregulated in class | OA |
| GeneSet | GOMF\_PEPTIDASE\_ACTIVATOR\_ACTIVITY\_INVOLVED\_IN\_APOPTOTIC\_PROCESS |
| Enrichment Score (ES) | 0.37942496 |
| Normalized Enrichment Score (NES) | 1.2112849 |
| Nominal p-value | 0.21158129 |
| FDR q-value | 0.5147073 |
| FWER p-Value | 1.0 |
Table: GSEA Results Summary

  

Fig 1: Enrichment plot: GOMF\_PEPTIDASE\_ACTIVATOR\_ACTIVITY\_INVOLVED\_IN\_APOPTOTIC\_PROCESS      
 Profile of the Running ES Score & Positions of GeneSet Members on the Rank Ordered List

  

| SYMBOL | TITLE | RANK IN GENE LIST | RANK METRIC SCORE | RUNNING ES | CORE ENRICHMENT || 1 | NLRP1 | NLR family pyrin domain containing 1 [Source:HGNC Symbol;Acc:HGNC:14374] | 200 | 0.819 | 0.0771 | Yes |
| 2 | CTSC | cathepsin C [Source:HGNC Symbol;Acc:HGNC:2528] | 227 | 0.801 | 0.1657 | Yes |
| 3 | CASP1 | caspase 1 [Source:HGNC Symbol;Acc:HGNC:1499] | 431 | 0.694 | 0.2284 | Yes |
| 4 | BEX3 | brain expressed X-linked 3 [Source:HGNC Symbol;Acc:HGNC:13388] | 438 | 0.690 | 0.3060 | Yes |
| 5 | EBAG9 | estrogen receptor binding site associated antigen 9 [Source:HGNC Symbol;Acc:HGNC:3123] | 493 | 0.662 | 0.3767 | Yes |
| 6 | PYCARD | PYD and CARD domain containing [Source:HGNC Symbol;Acc:HGNC:16608] | 1164 | 0.484 | 0.3794 | Yes |
| 7 | CTSH | cathepsin H [Source:HGNC Symbol;Acc:HGNC:2535] | 1803 | 0.384 | 0.3733 | No |
| 8 | CASP8AP2 | caspase 8 associated protein 2 [Source:HGNC Symbol;Acc:HGNC:1510] | 3186 | 0.232 | 0.2923 | No |
| 9 | MAL | "mal, T cell differentiation protein [Source:HGNC Symbol;Acc:HGNC:6817]" | 3265 | 0.224 | 0.3117 | No |
| 10 | BAD | BCL2 associated agonist of cell death [Source:HGNC Symbol;Acc:HGNC:936] | 3788 | 0.178 | 0.2913 | No |
| 11 | RPS27L | ribosomal protein S27 like [Source:HGNC Symbol;Acc:HGNC:18476] | 3882 | 0.166 | 0.3029 | No |
| 12 | BCL2L13 | BCL2 like 13 [Source:HGNC Symbol;Acc:HGNC:17164] | 5322 | 0.046 | 0.1964 | No |
| 13 | RACK1 | receptor for activated C kinase 1 [Source:HGNC Symbol;Acc:HGNC:4399] | 5983 | -0.003 | 0.1455 | No |
| 14 | ATP2A3 | ATPase sarcoplasmic/endoplasmic reticulum Ca2+ transporting 3 [Source:HGNC Symbol;Acc:HGNC:813] | 6559 | -0.046 | 0.1061 | No |
| 15 | ST20 | suppressor of tumorigenicity 20 [Source:HGNC Symbol;Acc:HGNC:33520] | 10517 | -0.375 | -0.1586 | No |
| 16 | NOD1 | nucleotide binding oligomerization domain containing 1 [Source:HGNC Symbol;Acc:HGNC:16390] | 10953 | -0.424 | -0.1444 | No |
| 17 | NKX3-1 | NK3 homeobox 1 [Source:HGNC Symbol;Acc:HGNC:7838] | 11348 | -0.478 | -0.1208 | No |
| 18 | APAF1 | apoptotic peptidase activating factor 1 [Source:HGNC Symbol;Acc:HGNC:576] | 12812 | -0.993 | -0.1221 | No |
| 19 | CARD8 | caspase recruitment domain family member 8 [Source:HGNC Symbol;Acc:HGNC:17057] | 12873 | -1.139 | 0.0021 | No |
Table: GSEA details [plain text format]

  

Fig 2: GOMF\_PEPTIDASE\_ACTIVATOR\_ACTIVITY\_INVOLVED\_IN\_APOPTOTIC\_PROCESS      
 Blue-Pink O' Gram in the Space of the Analyzed GeneSet

  

Fig 3: GOMF\_PEPTIDASE\_ACTIVATOR\_ACTIVITY\_INVOLVED\_IN\_APOPTOTIC\_PROCESS: Random ES distribution      
 Gene set null distribution of ES for **GOMF\_PEPTIDASE\_ACTIVATOR\_ACTIVITY\_INVOLVED\_IN\_APOPTOTIC\_PROCESS**
